# Post-Influenza Vaccine Subdeltoid bursitis

**DOI:** 10.7759/cureus.10764

**Published:** 2020-10-02

**Authors:** Marenda Jenkins, David Rupp, Lynne J Goebel

**Affiliations:** 1 Internal Medicine and Geriatrics, Joan C. Edwards School of Medicine at Marshall University, Huntington, USA; 2 Family and Community Medicine, Joan C. Edwards School of Medicine at Marshall University, Huntington, USA

**Keywords:** shoulder injury, technique, bursitis, subdeltoid bursitis, subacromial bursitis, vaccine administration, influenza vaccine, complications, side effects, vaccination

## Abstract

A 61-year-old female presented three months following an influenza vaccination with ongoing pain since the injection and was diagnosed by MRI with subdeltoid bursitis. The patient received a short course of prednisone and several months of physical therapy before returning to pain-free function. The primary reason for injection-related subdeltoid bursitis is improper administration. It is a preventable issue, and improved training of healthcare workers on proper vaccine administration may decrease its occurrence.

## Introduction

Influenza vaccine can routinely cause some pain and swelling in the shoulder, but it usually resolves a few days after vaccination. Shoulder injury related to vaccine administration (SIRVA) is a serious albeit rare complication of vaccination [1,2]. According to the Vaccine Adverse Event Reporting System, 731 patients self-reported either bursitis or bursa infection from vaccination in the 30 years between 1990 and 2020, a small number of cases considering that over 155 million doses are administered annually in the United States [3,4]. Current guidelines are to administer the vaccine three finger widths below the acromion or in the lower two-thirds of the deltoid muscle in adults [5].

Patients with SIRVA typically report the injection site to be noticeably higher than with previous vaccinations [2,6,7]. The effects can last months or even be permanent in some cases. Most commonly, patients with subdeltoid bursitis complain of pain and reduced function of the affected arm. The treatment for these patients may include medication, physical therapy and sometimes surgery. Some, but not all, treated cases result in an eventual painless return of function.

The mainstay of prevention for this injury is to ensure technically proper vaccine administration. We present this case of bursitis following influenza vaccination to remind healthcare professionals of the proper technique required to prevent injection-related morbidity.

## Case presentation

**Figure 1:**
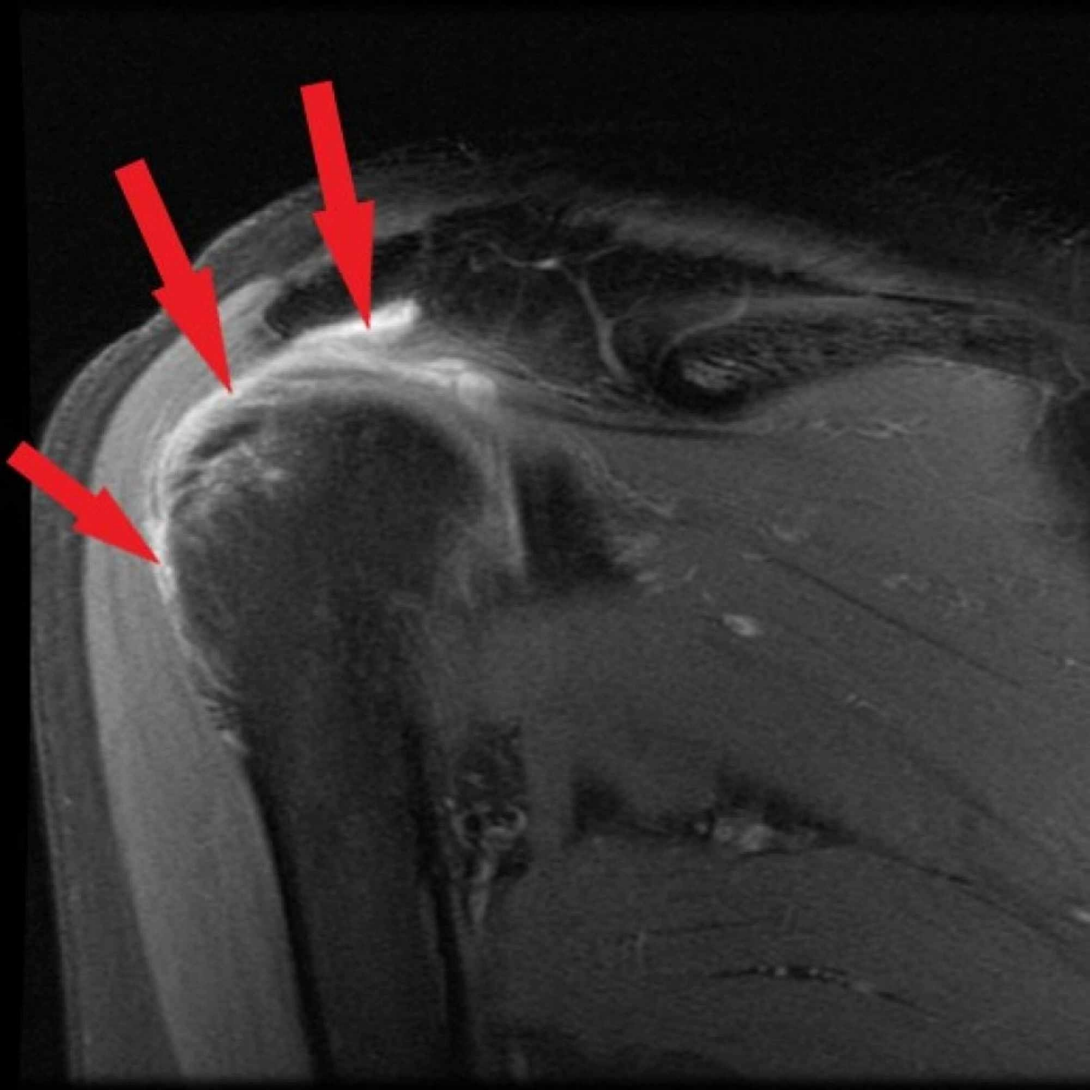
MRI left shoulder MRI left shoulder with arrows showing excess fluid in the subdeltoid bursae indicating bursitis

A 61-year-old female presented with ongoing shoulder pain three months after receiving influenza vaccination. Range of motion test revealed guarding with adduction/external rotation and pain with internal rotation. Neer test was positive, and the Hawkins test was negative. Strength of the rotator cuff muscles was preserved. An X-ray showed a type 2 acromion with a concave curved undersurface without other pathology, and the patient was prescribed meloxicam. MRI supported a diagnosis of subdeltoid bursitis (Figure 1).

The patient was treated with a short course of prednisone and physical therapy resulting in pain-free function after six months. 

## Discussion

Our patient had an injury after vaccination, possibly from her vaccine being administered too high. Subdeltoid bursitis, also known as subacromial bursitis, can result when the injection of vaccine is higher than the recommended three fingerbreadths below the acromion or if the injection is too deep going through the muscle and into the bursa. It is now considered a known but infrequent cause of vaccine-related morbidity [6,7]. Bodor and Montalvo looked at the position of the subdeltoid bursae in adults and found that it ranges from 3-6 cm past the acromion at a depth of 0.8-1.6 cm [8]. To ensure that the vaccine is injected into the muscle, the Centers for Disease Control and Prevention developed guidelines for proper administration [4,5]. Intramuscular injections are administered at a 90-degree angle into the deltoid muscle of the upper arm for adults, roughly three finger widths below the acromion. The needle gauge is to be 22-25 gauge with the determining factor of which to use based on the person’s size of the muscle, thickness of adipose tissue at the injection site, and the volume of material to be administered. The needle lengths to be used are based on administration technique of either the skin stretched flat (5/8 inch) or the subcutaneous and muscle tissue being bunched (1 inch). Needle lengths also vary by patient weight (patients weighing <130 lbs (60 kg), 5/8 inch; 130-152 lbs (60-70 kg), 1 inch; women weighing 152-200 lbs (70-90 kg) and men weighing 152-260 lbs (70-118 kg), 1-1.5 inch; women weighing >200 lbs (>90 kg) and men weighing >260 lbs (>118 kg), 1.5 inch). Inappropriate needle length could be responsible for an injection going too deep, especially in the case of people with little muscle mass.

Subdeltoid bursitis results in pain at rest that increases with use [9]. The differential diagnosis includes polymyalgia rheumatica, rotator cuff tear or tendonitis, and adhesive capsulitis. Pinpointing the diagnosis is difficult by physical examination alone. Our patient had a positive Neer test, or pain with passive flexion at the shoulder, which could indicate impingement or rotator cuff tendonitis. However, the Hawkins test, internal rotation of the shoulder with the arm at 90 degrees of flexion, was negative for impingement. The history of pain within two days of the vaccination is key to the clinical diagnosis. A corticosteroid injection can be helpful in that a complete response is unlikely to be seen with rotator cuff tear or adhesive capsulitis and patients with polymyalgia rheumatica may have a more generalized improvement in function of both shoulders with steroids. Bursitis resulting from improper vaccination is treated like any other inflammatory shoulder injury initially with analgesics, corticosteroid injections, and physical therapy. Our patient and her physician chose orally administered steroids to avoid any potential risks from further injections. In patients who do not respond to conservative treatment, imaging with MRI may be needed to confirm the diagnosis. Up to one-third of patients with subdeltoid bursitis require surgery, with many experiencing residual symptoms. 

Martin Arias et al. reviewed the international literature and the Spanish Pharmacovigilance System database and found 45 cases of a vaccine-related shoulder injury that included subdeltoid bursitis, osteonecrosis, frozen shoulder, septic shoulder and nerve palsies [10]. They reported vaccines other than influenza causing shoulder injury including pneumococcal polysaccharide, human papillomavirus, diphtheria-tetanus-poliomyelitis and hepatitis A although most (28, 62%) were associated with an influenza vaccine. The mean age of those affected was 53.6, with 71.1% women. Thirteen cases included in the Martin Arias series included vaccine-related shoulder injury reported to the Vaccine Injury Compensation Program (VICP) occurring over four years [11]. Most people had symptoms either immediately (7, 54%) or within 24 hours (5, 39%). Almost half of the people with shoulder injury reported the vaccine being given too high, similar to our patient. In this series, more than two-thirds of patients had some residual symptoms. VICP added subdeltoid bursitis as a compensable condition in 2017 with a requirement that symptoms begin by two days after vaccination.

Many people associate the influenza vaccine specifically with the cause of shoulder injury; however, this may be because it is an annual vaccine with more opportunities for improper administration. It is further hypothesized that if the vaccine is injected into the bursa, antibodies from prior vaccines present in synovium could cause inflammation leading to a longer duration of pain and this may be more likely to happen with influenza vaccine since it is repeated yearly [8].

A retrospective cohort study by Hesse et al. of almost three million people who received the influenza vaccine revealed 16 cases of subdeltoid bursitis occurring within two days of vaccine administration [12]. The mean age was 57.5, with over two-thirds being women. They reported a risk of 7.78 excess cases of subdeltoid bursitis per million people vaccinated. Fourteen of the 16 cases still had symptoms for a median of 386 days of follow up. Only two of the patients reported improper vaccination techniques- one thought it was too high and one too deep.

Most vaccines are administered by nurses, although vaccination by pharmacists is becoming more popular as payment for some vaccines is provided by the patient’s prescription insurance. In the large cohort study by Hesse et al., most of the vaccines causing bursitis were administered by medical assistants who have less training than nurses [12]. However, this may be due to medical assistants administering more vaccinations than nurses who have other duties and not that the medical assistants are more likely to give the vaccine incorrectly. Current regulations require nurses and medical assistants to receive a specified number of continuing education hours to maintain licensure. The number of hours varies according to provider type and state of licensure. To our knowledge, there is no specific requirement for hours dedicated to immunization administration, and perhaps this needs to be reviewed [13]. 

## Conclusions

Our patient had subdeltoid bursitis after receiving an influenza vaccine, a rare but now accepted complication possibly due to improper administration. Identification of the correct landmarks and use of appropriate needle length is critical to prevent the inadvertent introduction of the vaccine into the subdeltoid bursae, which may lead to debilitating complications. The importance of proper injection technique avoiding the upper third of the deltoid muscle and using appropriate length needles for the patient’s body habitus should be emphasized to healthcare providers in their training and continuing education.

## References

[1] Bancsi A, Houle SKD, Grindrod KA (2019). Shoulder injury related to vaccine administration and other injection site events. Can Fam Physician.

[2] Cook IF (2103). Subdeltoid/subacromial bursitis associated with influenza vaccination. Human Vaccin Immunother.

[3] (2020). Vaccine Adverse Event Reporting System. Vaccine Adverse Event Reporting System.

[4] (2020). Summary of the 2017-2018 Influenza Season. Centers for Disease Control and Prevention. https://tools.cdc.gov/medialibrary/index.aspx#/media/id/309789.

[5] (2020). ACIP Vaccine Administration Guidelines for Immunization. https://www.cdc.gov/vaccines/hcp/acip-recs/general-recs/administration.html.

[6] Cross GB, Moghaddas J, Buttery J, Ayoub S, Korman TM (2016). Don’t aim too high: avoiding shoulder injury related to vaccine administration. Aust Fam Physician.

[7] Cook IF (2015). Best vaccination practice and medically attended injection site events following deltoid intramuscular injection. Hum Vaccin Immunother.

[8] Bodor M, Montalvo E (2006). Vaccination-related shoulder dysfunction. Vaccine.

[9] Todd DJ (2020). Bursitis: An overview of clinical manifestations, diagnosis, and management. UpToDate. https://www.uptodate.com/contents/bursitis-an-overview-of-clinical-manifestations-diagnosis-and-management.

[10] Martin Arias LH, Sanz Fadrique R, Sainz Gil M, Salgueiro-Vazquez ME (2017). Risk of bursitis and other injuries and dysfunctions of the shoulder following vaccinations. Vaccine.

[11] Atanasoff S, Ryan T, Lightfoot R, Johann-Liang R (2010). Shoulder injury related to vaccine administration. Vaccine.

[12] Hesse EM, Navarro RA, Daley MF (2020 ). Risk for subdeltoid bursitis after influenza vaccination: A population-based cohort study. Ann Intern Med.

[13] (2020). West Virginia RN Board continuing education requirements. https://wvrnboard.wv.gov/education/Pages/default.aspx.

